# Identification of lectin counter-receptors on cell membranes by proximity labeling

**DOI:** 10.1093/glycob/cwx063

**Published:** 2017-07-28

**Authors:** Gang Wu, Manjula Nagala, Paul R Crocker

**Affiliations:** 1 Division of Cell Signalling and Immunology, School of Life Sciences, University of Dundee, The Wellcome Trust Building, Dow Street, Dundee DD1 5EH, UK

**Keywords:** glycophorin, lectin counter-receptor, proximity labeling, sialoadhesin

## Abstract

Lectin–glycan interactions play important roles in many biological systems, but the nature of glycoprotein counter-receptors expressed on cell membranes is often poorly understood. To help overcome this problem, we developed a method based on proximity labeling technology. Using a peroxidase-coupled lectin, addition of H_2_O_2_ and tyramide-biotin substrates leads to generation of short-range biotin radicals that biotinylate proteins in the immediate vicinity of the bound lectin, which can subsequently be identified. As a proof-of-principle, sialoadhesin-horseradish peroxidase-human IgG1 Fc recombinant protein constructs were precomplexed with anti-Fc antibodies, bound to human erythrocytes and reacted with H_2_O_2_ and tyramide-SS-biotin. The erythrocyte membrane protein with strongest biotinylation was identified as glycophorin A, in agreement with early studies using lectin overlay and reglycosylation approaches. As a further test of the method, the plant lectin MAL II was conjugated with horseradish peroxidase and used in proximity labeling of human erythrocytes. Glycophorin A was again selectively labeled, which is consistent with previous reports that MAL II has high affinity for glycophorin. This method could be applied to other lectins to identify their membrane counter-receptors.

## Introduction

Membrane and secreted proteins are commonly modified by heterogeneous N- and O-glycosylation. The glycans attached to proteins can be recognized by lectins, which play important roles in infection, immune responses, cell migration and activation (Sharon and Lis 2004). The first lectin was discovered more than 100 years ago (Lee and Lee 1995; Sharon and Lis 2004) and a large number of lectins have since been identified. Using defined glycans and new technologies such as glycan arrays (Rillahan and Paulson 2011), we now have a good understanding of their preferred oligosaccharide ligands, but less is known about the nature of glycoprotein counter-receptors which carry these ligands (Crocker and Feizi 1996). As a result of the requirement for multivalency and higher order clustering for lectin binding, affinity isolation techniques commonly used to study protein–protein interactions may not be suitable for studying lectin–glycoprotein interactions. For example, the detergents required for solubilization of membranes are likely to disrupt the organization and topography of membrane glycoproteins that could be critical for lectin recognition. In addition, affinity isolation with immobilized lectins could select artificially for glycoproteins through avidity effects or lead to interactions with intracellular glycoproteins that are not normally found at the cell surface. To circumvent these issues, it would be desirable to identify lectin counter-receptors at the cell surface of intact living cells.

Proximity labeling technologies have been used recently to investigate protein–protein interactions in intact cells (Rees et al. 2015a). In this technique, an enzyme such as a promiscuous form of a biotin ligase, BirA* (Roux et al. 2012) or a plant peroxidase (Li et al. 2014) is conjugated to a bait protein. When the bait binds to its target protein in the presence of enzyme substrates, these are converted into reactive species, which are only stable over short ranges, leading to labeling of proteins in the vicinity of the bait. The tagged proteins can then be enriched by affinity chromatography and identified by mass spectrometry or other suitable techniques. These proximity labeling technologies provide possible ways to identify lectin counter-receptors on intact cell membranes of living cells. For soluble lectins which usually exist as oligomeric complexes, such as the collectins or those isolated from plants, direct conjugation of the lectins with BirA* or peroxidase could be used to identify lectin counter-receptors. For membrane lectins, conversion to a soluble form such as the commonly used IgG Fc chimeras, followed by complex formation with anti-IgG Fc polyclonal antibody allows measurement of their interactions with glycan ligands at the cell surface (Redelinghuys et al. 2011; Kidder et al. 2013; Naito-Matsui et al. 2014). As a result, in vitro complexes of lectin-peroxidase-Fc or lectin-BirA*-Fc fusion proteins could be generated for proximity labeling using polyclonal anti-IgG Fc.

In this study, we developed a proximity labeling method to identify counter-receptors on cell membranes for both membrane and soluble lectins based on horseradish peroxidase (HRP)-catalyzed biotinylation. This proximity labeling method utilizes tyramide-SS-biotin as an HRP substrate, which turns into short lived tyrosyl radicals that can couple to tyrosine residues on proximal proteins (Rees et al. 2015b). As a model system, we used human erythrocytes which are heavily sialylated and are recognized by various soluble and membrane-bound lectins. Sialoadhesin (Sn, Siglec-1, CD169) was selected as an example of a monomeric membrane lectin since it has been well characterized in our laboratory in erythrocyte binding studies. MAL II was selected as an example of a soluble lectin as it is commercially available as a biotin conjugate that can be readily coupled to streptavidin-HRP for proximity labeling experiments.

## Results

### Proximity labeling of erythrocyte glycoproteins using Sn chimeric proteins

**Fig. 1.**
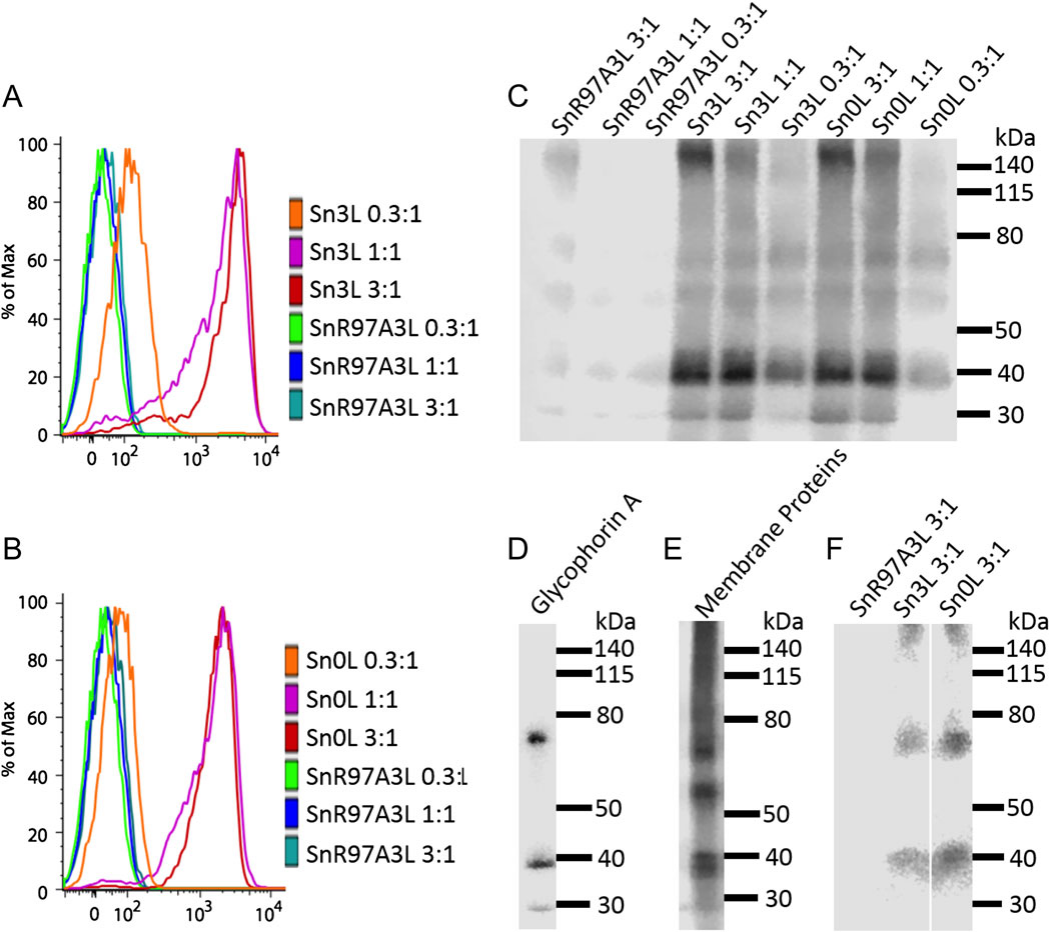
The binding and biotinylation activities of Sn-HRP-Fc chimeras. Sn3L and Sn0L denotes there are 3 and 0 linkers (GSGGGGSGGG) between the Sn and HRP respectively. SnR97A3L has a R97A mutation in Sn of the Sn3L chimera and was used as a negative control. For the binding assay, the concentration of each chimera was kept constant at 2.5 μg/mL, with addition of 7.5, 2.5 and 0.8 μg/mL FITC-conjugated goat anti-human IgG Fc to prepare immune-complexes at ratios of 3:1, 1:1 and 0.3:1 anti-Fc:Sn chimera, respectively, which are shown after the names of Sn chimeras in the figure. The binding to human erythrocytes was analyzed by flow cytometry. For the biotinylation assay, the concentration of each chimera was kept constant at 10 μg/mL, and complexes at ratios of 3:1, 1:1 and 0.3:1 anti-Fc-FITC:Sn-HRP-Fc chimera were prepared, which are shown after the names of Sn chimeras in the figure. (**A**) Binding of Sn3L to erythrocytes. (**B**) Binding of Sn0L to erythrocytes. (**C**) Biotinylation of erythrocytes by Sn chimeras. Cells were lysed and blotted with streptavidin-HRP. (**D**) Erythrocyte lysate blotted with anti-glycophorin A. (**E**) Total erythrocyte surface proteins labeled using sulfo-NHS-SS-biotin and the cell lysate was blotted by streptavidin-HRP. (**F**) Proteins biotinylated using Sn-HRP-Fc chimeras were pulled down with streptavidin magnetic beads, eluted by reducing LDS sample buffer and blotted with anti-glycophorin A. This figure is available in black and white in print and in color at *Glycobiology* online.

For the initial proximity labeling experiments, Sn-horseradish peroxidase-human IgG1 Fc (Sn-HRP-Fc) chimeras were produced using a baculovirus expression system (Supplementary Figure S1). In order to determine if a linker region between Sn and HRP is required to provide flexibility for Sn to bind and for HRP to label Sn counter-receptors efficiently, we generated two types of Sn-HRP-Fc chimera named Sn3L and Sn0L (Supplementary Figure S1). Sn3L has 3 GSGGGGSGGG linkers and surrounding sequences, with a total length up to ~17 nm between Sn and HRP (calculated on the basis that the maximum linear dimension of a polypeptide with *n* residues is *n* × 3.6 Angstroms) (Oesterhelt et al. 2000), whereas Sn0L lacks linkers. For the preparation of multimers, each chimera concentration was kept constant, with addition of different amount of goat anti-human IgG Fc to prepare immune-complexes at ratios of 3:1, 1:1 and 0.3:1 anti-Fc:Sn chimera, respectively. Flow cytometry analysis revealed that the binding of precomplexed Sn-HRP-Fc chimeras was not greatly affected by the presence of linkers, as both the Sn0L and Sn3L demonstrated similar binding profiles to erythrocytes (Figure 1A, B), with the strongest binding observed at the 1:1 and 3:1 ratios of FITC-anti-Fc:Sn-HRP-Fc (Figure 1A, B). As expected, the negative control protein, SnR97A3L, showed no binding activity at all ratios tested (Figure 1A, B).

In the proximity labeling experiments, strong biotin labeling of a 40 kDa band was observed at the 1:1 and 3:1 ratios of anti-Fc:Sn-HRP-Fc that was not seen with the SnR97A3L control protein (Figure 1C). Further evidence for specific labeling of the 40 kDa band was seen using α-methyl-NeuAc as a competitive inhibitor of Sn binding and biotinylation of erythrocytes (Supplementary Figure S2).

Previous studies indicated that Sn binds to glycophorin on human erythrocytes (Crocker et al. 1991). Glycophorin A is the major glycophorin on erythrocytes and the monomeric form has an apparent molecular mass close to 40 kDa (Chasis and Mohandas 1992). We therefore verified if this biotinylated 40 kDa band corresponds to glycophorin A. Western blotting for glycophorin A using total erythrocyte lysates and streptavidin pulldowns of proximity-labeled material demonstrated that the 40 kDa band corresponded to monomeric glycophorin A (Figure 1D, F). A dimeric form of glycophorin A at ~80 kDa (Engelman et al. 1992) was also labeled by Sn-HRP-Fc proteins, most prominently at the 0.3:1 ratios of anti-Fc:Sn-HRP-Fc (Figure 1C). Higher molecular weight material above 140 kDa was also labeled (Figure 1C, F). This probably corresponds to biotinylated anti-Fc antibody and/or glycophorin A complexes resulting from the HRP catalyzed generation of di-tyrosine bonds, leading to intermolecular crosslinking (Minamihata et al. 2011). When the pattern of biotinylation in proximity labeling was compared with biotinylation of total membrane proteins using sulfo-NHS-biotin (Figure 1E), clear differences were seen, providing strong evidence that the proximity labeling leads to selective biotinylation of glycophorin A.

Given that many lectins are available as Fc-fusion proteins, we asked whether similar labeling patterns could be obtained using Sn-Fc protein complexed with HRP-conjugated anti-human Fc antibody. The 40 kDa band was strongly biotinylated but was absent from the SnR97A-Fc protein used under the same conditions (Supplementary Figure S3). These results indicate that very close proximity of HRP to the lectin binding site may not be crucial for successful proximity labeling of cell membranes. As an alternative strategy for proximity labeling that might more closely mimic cell-cell interactions, the Sn chimeras were immobilized on magnetic beads and then mixed with erythrocytes. Proximity labeling data revealed that the 40 kDa band was again preferentially biotinylated, using both Sn-HRP-Fc and Sn-Fc constructs (Supplementary Figure S4).

### Proximity labeling of erythrocyte glycoproteins using MAL II lectin

**Fig. 2.**
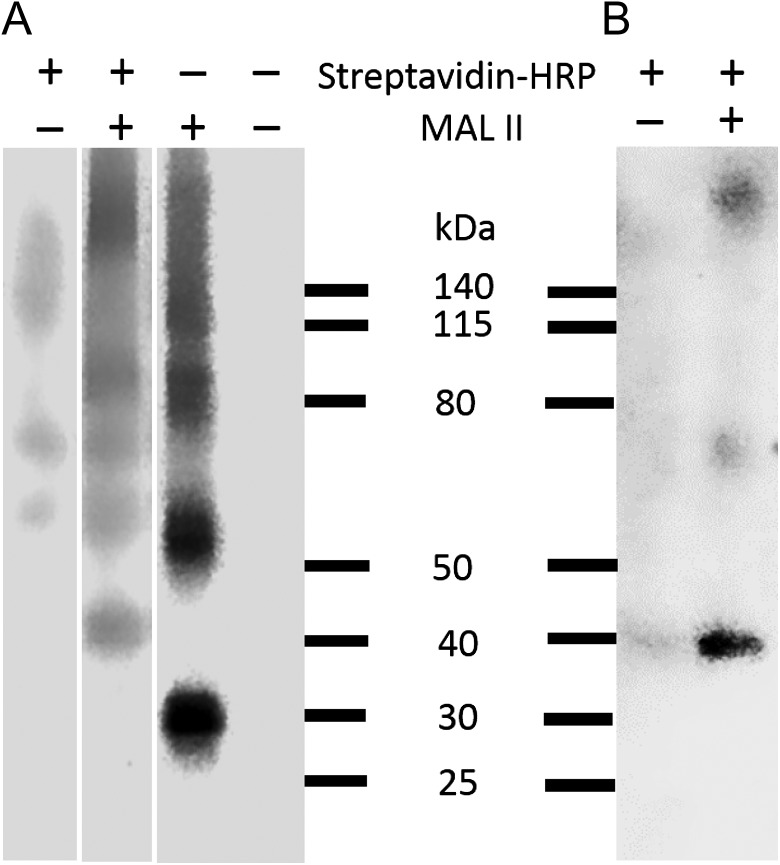
Biotinylation of human erythrocytes by HRP-conjugated MAL II lectin. (**A**) Erythrocytes were incubated with biotinylated MAL II followed by streptavidin-HRP. Erythrocytes mixed with the same amount of streptavidin-HRP only were used as a negative control. Erythrocytes incubated with MAL II only were used as an additional control to confirm the 40 kDa band was not from biotinylated MAL II. Biotinylated proteins were blotted with Streptavidin-HRP. (**B**) Biotinylated proteins were pulled down by streptavidin magnetic beads, eluted by LDS sample buffer with reducing reagent, and blotted with anti-glycophorin A.

MAL II is known to bind human erythrocytes and has a similar ligand binding preference to Sn, recognizing α2,3-linked sialic acids (Kawaguchi et al. 1974). The proximity labeling strategy was used to identify MAL II counter-receptors on erythrocytes. The cells were first incubated with biotinylated MAL II followed by streptavidin-HRP. The 40 kDa band corresponding to glycophorin A was biotinylated, but was not seen in control labeling experiments where the erythrocytes were mixed with either streptavidin-HRP or with biotinylated MAL II alone (Figure 2A). Pulldowns of proximity-labeled proteins demonstrated that the 40 kDa band corresponded to monomeric glycophorin A (Figure 2B). In the MAL II alone control, we saw strong biotinylated bands at 30 and 60 kDa (Figure 2A), which reflected the presence of the biotinylated MAL II. However, these two bands were not detected when streptavidin-HRP was present. Instead, proteins in the high molecular weight range (above 140 kDa) were observed, which could be due to HRP-induced crosslinking of MAL II-streptavidin-HRP complexes. Overall, the results showed specific labeling of glycophorin A by HRP-conjugated MAL II, which is consistent with previous reports that glycophorin A has a high affinity for MAL II (Konami et al. 1994; Geisler and Jarvis 2011).

## Discussion

Proximity labeling has been successfully applied in a growing number of studies to investigate protein–protein interactions (Roux et al. 2012; Li et al. 2014; Dingar et al. 2015; Rees et al. 2015b). However, applying proximity labeling to identify lectin counter-receptors is uniquely challenging due to the weak nature of the lectin–glycan interaction. In this study, we attempted to address the challenge and developed a method for lectin counter-receptor identifications on erythrocytes. Erythrocytes are easy to prepare and have been used for many years in hemagglutination assays to measure lectin binding activity (Sharon and Lis 2004). In the case of Sn and other Siglecs, human erythrocytes have been used extensively in a wide range of assays, as they carry high levels of NeuAc in both α2,3 and α2,6 linkages and can be experimentally modified to present specific homogenous sialic acid linkages, either through enzymatic reactions or ganglioside incorporation (Crocker et al. 1991; Kelm et al. 1994).

We verified our proximity labeling method by demonstrating that glycophorin A is a predominant counter-receptor for Sn and MAL II on human erythrocytes. Glycophorin A is a major glycoprotein of human erythrocytes and presents *O*-linked glycans terminating in α2,3-linked Neu5Ac, preferred glycan ligands for Sn and MAL II (Crocker et al. 1991; Geisler and Jarvis 2011). Our results are consistent with previous studies that implicated glycophorin A as a human erythrocyte counter-receptor for Sn and MAL II (Crocker et al. 1991; Konami et al. 1994; Geisler and Jarvis 2011). This method could be applied to identify unknown lectin membrane counter-receptors on other cells. In that case, the biotinylated proteins could be pulled down by streptavidin beads and the counter-receptors identified by quantitative proteomics, as has been well-documented in studies investigating protein–protein interactions by proximity labeling (Rees et al. 2015a; Kim and Roux 2016).

A promiscuous form of a biotin ligase, BirA*, is another enzyme commonly used for proximity labeling (Roux et al. 2012). By releasing activated biotin species, it labels lysine and arginine residues, which are more commonly found in proteins than tyrosine residues. In our initial experiments, we generated Sn-BirA*-Fc fusion proteins with 3 linkers and 0 linkers to develop a complementary proximity labeling system. Although the enzyme was active and both chimeras showed strong binding to erythrocytes, we failed to detect biotinylation of membrane proteins (unpublished observations), probably due to the shorter range of BirA*-activated biotin species compared to HRP-generated products (Rees et al. 2015b).

Overall, the proximity labeling method developed here has the potential to be applied to many other soluble and membrane-bound lectins in order to identify their membrane counter-receptors. This approach could provide new insights into the molecular targets and biological functions of lectins, including those expressed in the immune system and involved in cellular and pathogen interactions and those expressed by microorganisms and important in pathogenicity and host invasion.

## Materials and methods

### Materials

Sn-HRP-Fc DNA constructs were synthesized by GenScript (GenScript HK Limited). The Sn-Fc chimeras (Crocker et al. 1999) and Sf9 cells were from laboratory stocks. Sf-900 II serum free medium, FetalClone II serum, Dynabeads^®^ MyOne™ Streptavidin C1 magnetic beads, sulfo-NHS-SS-biotin, NuPAGE^®^ sodium dodecyl sulphate-polyacrylamide gel electrophoresis (SDS-PAGE) gels, MOPS running buffer, LDS sample buffer, sample reducing agent, and biotinylated goat anti-human IgG Fc were from Thermo Fisher Scientific (Paisley, UK). Tyramine hydrochloride, hemin, protein A-Sepharose^®^4B, FITC-conjugated goat anti-human IgG Fc, HRP-conjugated rabbit anti-mouse IgG and HRP-conjugated streptavidin were from Sigma (Dorset, UK). HRP-conjugated goat anti-human IgG Fc was from Abcam (Cambridge, UK). Mouse anti-human glycophorin A (Clone: BRIC 163) was from IBGRL Research Products (Bristol, UK). Biotinylated MAL II was from Vector Laboratories (Peterborough, UK). Complete™, Mini, EDTA-free protease inhibitor cocktail was from Roche (Boehringer, UK). Human erythrocytes were collected from volunteer donors under human subject protocols approved by the local ethics committee at the University of Dundee.

### Designing, expression and purification of Sn-HRP-Fc chimeras

The first 3 N-terminal Ig-like domains of Sn were selected for construct design. The Sn signal peptide was substituted by honey bee melittin signal peptide to enhance protein secretion. The DNA coding the honey bee melittin signal peptide, Sn, 3 GSGGGGSGGG linkers, HRP, and IgG Fc was cloned into pFastBac™1 as shown in Supplementary Figure S5. Further endonuclease restriction sites were added to make the construct versatile (Supplementary Figure S5). Sn in the construct can be changed for another lectin by cloning using *Rsr*II and *Eco*RI sites, HRP can be replaced by a promiscuous form of the biotin ligase BirA* through *Pfl*MI sites, and the number of linkers can be changed using *Kpn*I, *Sac*I or *Sal*I sites. L234A L235A mutations were introduced to IgG Fc to reduce its binding to Fc receptors (Radaev et al. 2001). R97A mutation was introduced to Sn as a negative control. Sn3L, SnR97A3L and Sn0L were expressed by Sf9 cells grown in Sf-900 II serum free medium, according to the instructions of Bac-to-Bac^®^ Baculovirus Expression System (Thermo Fisher Scientific). Hemin was added to the cell culture to a final concentration of 0.09 μM to increase the HRP activity of the chimeras (Segura et al. 2005). The proteins were purified by Protein A-Sepharose^®^ 4B and the purity was checked by SDS-PAGE.

### Flow cytometry analysis

About 2 × 10^6^ erythrocytes were suspended in 100 μL phosphate-buffered saline (PBS) containing 1% FetalClone II serum, 2.5 μg/mL Sn3L or Sn0L chimera, and varying concentrations of FITC-conjugated goat anti-human IgG Fc at 7.5, 2.5 and 0.8 μg/mL to prepare immune-complexes with the ratios of 3:1, 1:1 and 0.3:1 of anti-Fc to Sn chimera, respectively. After 1 h incubation on ice, the nonbound immune-complexes were washed out by centrifugation and the cells were re-suspended in 1 mL PBS containing 1% FetalClone II serum for flow cytometry analysis. SnR97A3L was used as a negative control. The data were analyzed by FlowJo.

### Proximity labeling of erythrocyte glycoproteins using complexes of Sn chimeric proteins in solution

For Sn3L- and Sn0L-guided proximity labeling, 7 × 10^6^ human erythrocytes were suspended in 144 μL PBS containing 1% FetalClone II serum, 10 μg/mL Sn chimera and varying concentrations of FITC-conjugated goat anti-human IgG Fc at 30, 10 and 3.3 μg/mL to prepare immune-complexes with the ratios of 3:1, 1:1 and 0.3:1 of anti-Fc to Sn chimera, respectively. The cells were incubated on ice for 50 min then at room temperature for 10 min. 1.5 μl 12 mM tyramide-SS-biotin in dimethylformamide, synthesized according to a previous report (Hopman et al. 1998), and 15 μL 0.3% H_2_O_2_ solution in PBS were added and incubated at room temperature for 5 min. A 1 mL ice-cold quenching buffer (1% FetalClone II serum, 100 U/mL catalase in PBS) was added and the cells were washed twice with quenching buffer and once with ice-cold PBS containing 1% FetalClone II serum. SnR97A3L was used as a negative control following the same protocol. For Sn-Fc-chimera-guided proximity biotinylation, the same method was used, except that 10 μg/mL Sn-Fc and 10 μg/mL HRP-conjugated anti-Fc were mixed to prepare Sn-Fc-anti-Fc-HRP complexes prior to addition of substrates.

### Proximity labeling of erythrocyte glycoproteins using Sn chimeras immobilized on beads

For Sn-HRP-Fc chimeras, 1 μg biotinylated anti-Fc protein was mixed with 10 μL streptavidin magnetic beads and incubated at 4°C overnight. The beads were then washed with PBS containing 1% FetalClone II serum and incubated with 1 μg Sn3L or Sn0L protein at 4°C for 2 h. Unbound Sn chimera was washed out with PBS containing 1% FetalClone II serum. For Sn-Fc chimeras, 1 ug Sn-Fc was mixed with 1 ug HRP-anti-Fc in 100 μL PBS containing 1% FetalClone II serum, incubated on ice for 30 min, then mixed at 4°C for 2 h with 10 μL magnetic beads which were pre-coated with biotinylated goat anti-human Fc. Unbound was washed out with PBS containing 1% FetalClone II serum. The beads were incubated on ice for 50 min with 6 × 10^6^ erythrocytes in 90 μl PBS containing 1% FetalClone II serum, then at room temperature for 10 min. For biotinylation, 2 μL tyramide-SS-biotin and 10 μL 0.3% H_2_O_2_ in PBS were added and incubated at room temperature for 5 min. The reaction was quenched as described previously.

### Biotinylation of erythrocyte proteins using MAL II lectin and sulfo-NHS-SS-biotin

For biotinylation of human erythrocytes using MAL II, 3 × 10^6^ erythrocytes were incubated on ice for 1 h with 66 μL PBS containing 2.5 μg/mL biotinylated MAL II and 1% FetalClone II serum. After washing out the nonbound MAL II with 1% FetalClone II serum in PBS, the cells were resuspended in 66 μL streptavidin-HRP at 5 μg/mL, 1% FetalClone II serum in PBS, incubated on ice for 30 min, then at room temperature for 10 min. A 1.5 μL tyramide-SS-biotin and 7 μL 0.3% H_2_O_2_ solution were added and incubated at room temperature for 5 min. The reaction was quenched as described previously. Erythrocytes mixed with the same concentrations of either streptavidin-HRP or MAL II alone were used as negative controls. For the biotinylation of total surface proteins on erythrocytes, 3 × 10^6^ PBS washed erythrocytes were mixed with 80 μL cold PBS adjusted to pH 8.0 and 20 μL 10 mM sulfo-NHS-SS-biotin in PBS pH 8.0 and incubated at 4°C for 30 min. The reaction was terminated by washing the cells three times using ice-cold TBS.

### Western blotting

Erythrocytes were lysed in 20 mM Tris–HCl, 5 mM EDTA, 150 mM NaCl, 1% (v/v) Triton X-100, 0.1 M sodium thiocyanate, pH 8.0 supplemented with protease inhibitor cocktail. A 20 μL lysis buffer was added per 10^6^ cells. The cell lysates were loaded to 4–12% Bis–Tris gel. Each lane corresponds to 4 × 10^5^ erythrocytes. The proteins were separated by SDS-PAGE using MOPS running buffer. Proteins were transferred to PVDF membranes for western blotting using streptavidin-HRP for the detection of biotinylated proteins. Glycophorin A was detected on western blots using mouse anti-human glycophorin A followed by rabbit anti-mouse IgG-HRP.

### Streptavidin pulldowns of biotinylated proteins

A 25 μL streptavidin-HRP dynabeads were washed using PBS, mixed with 30 μL lysate and 70 μL cell lysis buffer, and incubated at 4°C overnight. The beads were then washed sequentially according to a previous report (Rees et al. 2015b) in wash buffer 1 (10 mM Tris–Cl, 1% (v/v) Triton X-100, 1 mM EDTA, 0.5% (w/v) SDS, 500 mM NaCl, 0.1 M sodium thiocyanate, pH 7.4), wash buffer 2 (10 mM Tris–Cl, 1% (v/v) Triton X-100, 1 mM EDTA, 0.5% (w/v) SDS, 0.1 M sodium thiocyanate, pH 7.4) and PBS at room temperature for 10 min each. The bound proteins were eluted by incubating with 1 × LDS reducing sample buffer at room temperature for 10 min. The eluted proteins were analyzed by western blotting. Each lane corresponds to 4 × 10^5^ erythrocytes.

## Supplementary data

Supplementary data is available at *Glycobiology* online.

## Funding

This work was supported by the Wellcome Trust [103744/Z/14/Z].

## Conflict of interest statement

None declared.

## Supplementary Material

Supplementary Data

## Abbreviations

Fc, human IgG1 Fc region; HRP, horseradish peroxidase; Sn, Sialoadhesin; Sn-Fc, Sialoadhesin-human IgG1 Fc region chimera; SnR97A-Fc, Sialoadhesin-human IgG1 Fc region chimera with R97A mutation on Sn; SnR97A3L, Sialoadhesin-horseradish peroxidase-human IgG1 Fc region chimera with 3 GSGGGGSGGG linkers and R97A mutation on Sn; Sn0L, Sialoadhesin-horseradish peroxidase-human IgG1 Fc region chimera with 0 GSGGGGSGGG linkers; Sn3L, Sialoadhesin-horseradish peroxidase-human IgG1 Fc region chimera with 3 GSGGGGSGGG linkers.
