# Supplementary material for: Identification of lectin counter-receptors on cell membranes by proximity labeling
Source: Glycobiology. 2017 Jul 28;27(9):800–5. doi: 10.1093/glycob/cwx063 (PMC5881670; doi:10.1093/glycob/cwx063)
Supplement: Supplementary Data [file cwx063supplementarydata.pdf]

Supplementary data

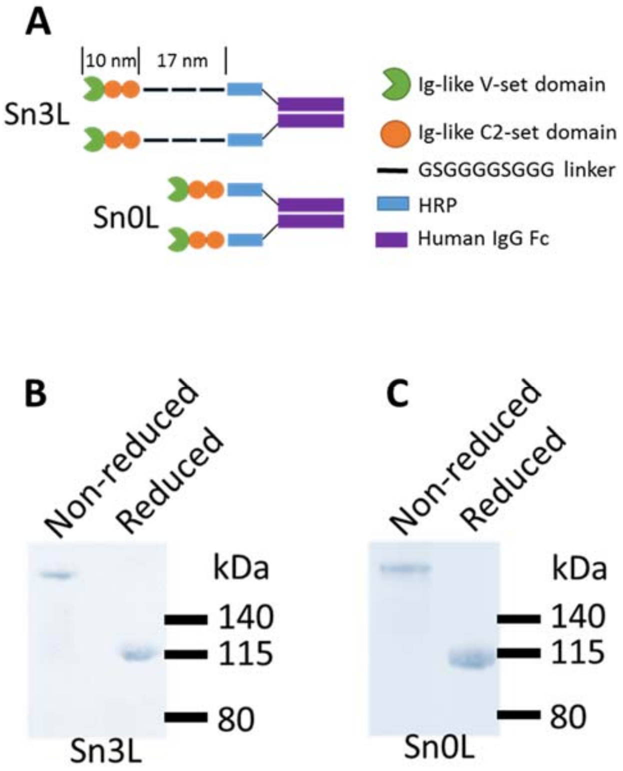

Supplementary Figure S1. A, Design of Sn-HRP-Fc chimera with 3 GSGGGGSGGG linkers (Sn3L) and 0 linkers (Sn0L) between Sn and HRP. **B and C**, the proteins were expressed using a baculovirus expression system, purified by protein A beads and analysed by non-reducing and reducing SDS-PAGE.

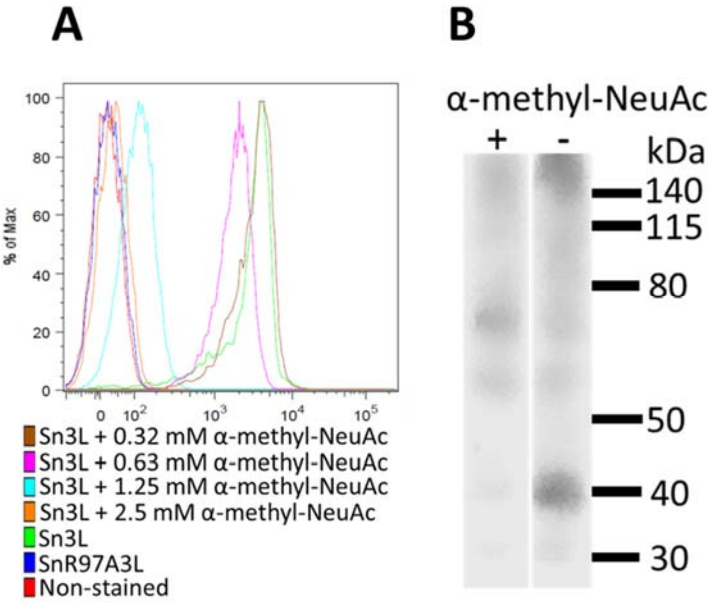

Supplementary Figure S2. A, inhibition of Sn chimera binding to erythrocytes by α-methyl-NeuAc. 2.5 µg/ml Sn chimera were immune-complexed with 2.5 µg/ml FITC-conjugated goat anti-human IgG Fc for the binding assay. SnR97A3L has a R97A mutation in Sn of the Sn3L chimera, which together with the non-stained erythrocytes served as negative controls. **B**, inhibition of biotinylation by α-methyl-NeuAc. 10 µg/ml Sn3L were immune-complexed with 10 µg/ml FITC-conjugated goat anti-human IgG Fc for binding and biotinylation of erythrocytes in the presence or absence of 5 mM α-methyl-NeuAc. The cell lysates were blotted with streptavidin-HRP.

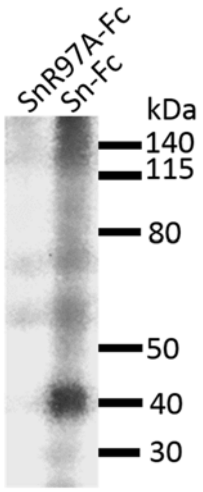

Supplementary Figure S3. Biotinylation of human erythrocytes using 10 µg/ml Sn-Fc complexed with 10 µg/ml HRP-conjugated goat anti-human IgG Fc. SnR97A-Fc was used as a negative control. The cell lysate was blotted with streptavidin-HRP.

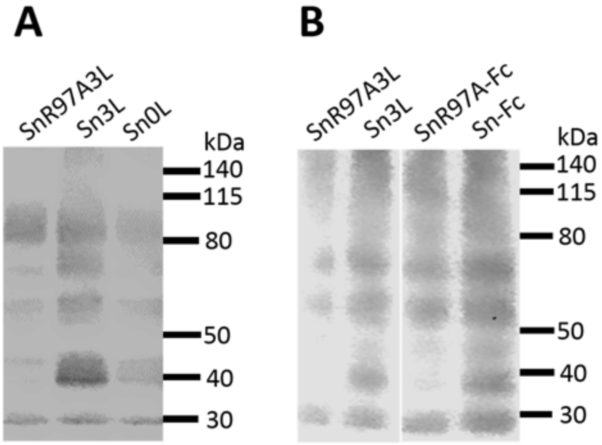

Supplementary Figure S4. Biotinylation of human erythrocytes using beads coated with Sn chimeras. In order to build SnR97A3L, Sn3L and Sn0L multimers on beads, biotinylated goat anti-human Fc was first immobilized on streptavidin magnetic beads, followed by SnR97A3L, Sn3L and Sn0L protein. In order to build SnR97A-Fc and Sn-Fc multimers on beads, HRP-conjugated goat anti-human IgG Fc was pre-mixed with Sn chimers to build in-solution multimers, which were then immobilized to magnetic beads pre-coated with biotinylated goat anti-human Fc. **A,** comparison of Sn3L and Sn0L on-bead biotinylation of erythrocytes. **B,** comparison of Sn3L and Sn-Fc on-bead biotinylation of erythrocytes.

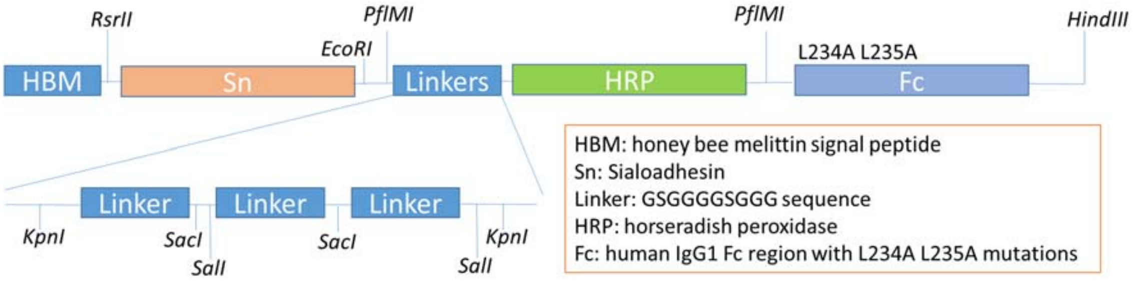

Supplementary Figure S5. Design of a versatile Sn-HRP-Fc construct in pFastBac™1. The first 3 N-terminal Ig-like domains of Sn were selected for designing the constructs. The Sn signal peptide was substituted by honey bee melittin signal peptide to enhance protein secretion. *RsrII* and *EcoRI* sites can be used to change Sn for other lectins of interest. *PflMI* sites can be used to change HRP for a promiscuous form of the biotin ligase BirA*. *KpnI*, *SacI* or *SalI* sites can be used to change the numbers of the linkers. L234A L235A mutations were introduced to IgG Fc to reduce its binding to Fc receptors.
